# Multiple imputation for estimation of an occurrence rate in cohorts with attrition and discrete follow-up time points: a simulation study

**DOI:** 10.1186/1471-2288-10-79

**Published:** 2010-09-03

**Authors:** Noémie Soullier, Elise de La Rochebrochard, Jean Bouyer

**Affiliations:** 1 INSERM, CESP Centre for research in Epidemiology and Population Health, U1018, Reproduction and Child Development Team, F-94276 Le Kremlin-Bicêtre, France; 2 Univ Paris-Sud, UMRS 1018, F-94276 Le Kremlin-Bicêtre, France; 3 INED, F-75020 Paris, France

## Abstract

**Background:**

In longitudinal cohort studies, subjects may be lost to follow-up at any time during the study. This leads to attrition and thus to a risk of inaccurate and biased estimations. The purpose of this paper is to show how multiple imputation can take advantage of all the information collected during follow-up in order to estimate the cumulative probability *P(E) *of an event *E*, when the first occurrence of this event is observed at *t *successive time points of a longitudinal study with attrition.

**Methods:**

We compared the performance of multiple imputation with that of Kaplan-Meier estimation in several simulated attrition scenarios.

**Results:**

In missing-completely-at-random scenarios, the multiple imputation and Kaplan-Meier methods performed well in terms of bias (less than 1%) and coverage rate (range = [94.4%; 95.8%]). In missing-at-random scenarios, the Kaplan-Meier method was associated with a bias ranging from -5.1% to 7.0% and with a very poor coverage rate (as low as 0.2%). Multiple imputation performed much better in this situation (bias <2%, coverage rate >83.4%).

**Conclusions:**

Multiple imputation shows promise for estimation of an occurrence rate in cohorts with attrition. This study is a first step towards defining appropriate use of multiple imputation in longitudinal studies.

## Background

Longitudinal studies are the most appropriate way of analysing the occurrence of an event *E *and of estimating its cumulative probability during follow-up *P(E)*. One of the most important drawbacks of such studies is that subjects may be lost to follow-up at any time point. These drop-outs accumulate over time and lead to attrition of the initial cohort, which has fewer and fewer participants as time goes by. Attrition can lead to biased and inaccurate estimations of *P(E) *[1], especially in classical complete-case analyses [2,3]. For example, in a UK cohort study of ocular outcome after premature birth, which involved 558 children born before 32 weeks of gestation in 1990-1991, abnormalities were more frequent among drop-outs, and complete-case analysis thus underestimated the prevalence of ocular abnormalities in the study population [4].

Survival analysis and Kaplan-Meier estimation are commonly used to examine time-to-event measurements [5,6]. This approach takes into account the fact that subjects are followed for different lengths of time. However, it assumes that censored patients (including patients lost to follow-up) would have the same probability of experiencing a subsequent event as non-censored patients. This assumption has been challenged in various fields [4,7,8]. One specific example, which motivated the present study, is the issue of *in vitro *fertilization (IVF) success rates [9]. In IVF cohorts, drop-out corresponds to treatment interruption, which is partly linked to a poor likelihood of success [10]. Couples who drop out therefore have a lower chance of success than couples who persevere [11]. The Kaplan-Meier approach tends to overestimate the IVF success rate, and alternative methods have recently been proposed: drop-outs are divided into two groups depending on the chances of success (poor/not poor) [12], or, equivalently, according to whether IVF treatment is interrupted for medical causes or not [13]. Couples with a poor chance of success will be considered as having a zero probability of subsequent success, whereas those with a good prognosis will have the same chance of subsequent success as those who persevere. This method makes it possible to take into account auxiliary information on the probability of success among drop-outs, through individual prognostic factors, considered in two groups. This idea may be developed by looking for ways of taking into account more of this auxiliary information. Multiple imputation is a good candidate approach in this setting.

Multiple imputation was developed thirty years ago [14] and is now used in observational as well as in randomized studies [15,16]. However, epidemiological applications have been limited [17]. Previous works have addressed the use of multiple imputation in longitudinal studies [18-20], but few focused on estimation of the occurrence rate at the end of the follow-up. This is a special issue, as it implies imputation of the missing covariates and outcomes. Hsu et al. developed a weighted Kaplan-Meier method to take into account dependant interval censoring in the estimation of a recurrence rate [21,22]. Our objective is similar. However, we preferred to use multiple imputation, which consists in replacing missing values with a set of plausible values, based on auxiliary information and which has the advantage of being routinely implemented and easily applicable. Moreover, IVF studies have a particular way of defining time, as each time point of the study is an IVF attempt. The length of time elapsing between the attempts is not considered. The method of Hsu et al. implied imputing midpoints of intervals and is therefore not appropriate here. Kaplan-Meier estimation was chosen for comparison with multiple imputation, because this method is often used to estimate cumulative success rates in IVF.

The purpose of this paper is to show how multiple imputation can take advantage of all information collected during follow-up. It focuses specifically on the estimation of a cumulative probability *P(E) *of an event *E*, when the first occurrence of this event is observed at *t *successive time points of a longitudinal study. The performance of multiple imputation was compared with that of the Kaplan-Meier approach by simulating several attrition scenarios.

## Methods

### Attrition types

Three types of missingness mechanisms can be distinguished: missing completely at random (MCAR), missing at random (MAR) and not missing at random (NMAR) [23]. If the probability of drop-out depends neither on missing data nor on observed data, then the data are MCAR. In this case attrition implies a loss of power, but no bias occurs in complete-case estimations. An example of MCAR situation in the context of IVF is the accidental loss of patient records. If, given the observed data, the probability of drop-out does not depend on missing data, then the data are MAR. Bias may arise but it can be minimized with appropriate methods, such as multiple imputation, that take into account available information explaining the missingness mechanism. As an example, suppose that records of patients older than 35 years are recorded separately (file A for patients younger than 35, and file B for patients older than 35). The situation is MAR if all the file A is available but a part of file B is accidentally lost. Finally, if the probability of drop-out depends on missing data or on other unobserved variables, then the data are NMAR. Bias due to attrition cannot be reduced in this case, and sensitivity analyses have to be conducted under various NMAR assumptions. An example is the situation in which patients are recorded differentially according to the outcome of the attempt: all patients with a delivery would be recorded, but only a part of those with pregnancy loss would. The present study focuses on MAR attrition.

### Simulation study

Data were simulated using SAS Software (SAS Institute Inc. 2004. SAS/STAT^® ^9.1 User's Guide. Cary, NC: SAS Institute Inc.).

The simulation process took place in three steps detailed below: first, a complete cohort was simulated, then attrition was added and samples were randomly drawn, and finally the different methods of estimation were applied to the samples with attrition.

### Complete cohort generation

A cohort of 100 000 subjects with four follow-up time points was simulated. Simulated data consisted in a binary outcome *E *representing occurrence of the event, and a continuous covariate *X *predictive of both occurrence and drop-out. Measures for subject *i *at time point *t *were named *E_it _*and *X_it_*. In our IVF motivating example, the outcome is delivery and the covariate is the age of the woman.

The covariate *X *was simulated Gaussian and the link between the successive time points was the following:

$$\begin{array}{l} X_{i1}\sim N(0,10)\\ \begin{array}{cc} X_{it}=\alpha_{it}+\beta_{it}\times X_{i(t-1)}+\varepsilon_{it}, & t=2,3,4,\varepsilon_{it}\sim N(0,s_{it}^{2}) \end{array} \end{array}$$

For simplicity, coefficients were taken as being equal to one: *α_it _*= *β_it _*= 1 and $s_{it}^{2}$ = 10.

The outcome *E_it _*was drawn from a Bernoulli distribution with a probability of occurrence of the event equal to *p_it_*, so that *p_it _*= *P(E_it _= 1)*. In order to make *X *a predictor of *E*, the value of *p_it _*was linked to the value of *X_it_*. The supposition was that two sub-populations with specific probabilities of occurrence of the event coexist in the population. To achieve this, subjects were divided into two categories *C_1 _*and *C_2 _*according to their *X_it _*values. The probabilities of occurrence of the event *p_it _*could differ according to the category. The threshold delimiting the two categories was the median of *X_1 _*(0.014). The same threshold was kept for all time points, i.e. *C_1 _*was defined as *X_it _*< 0.014 whatever the value of *t*. For a given category of subjects, the probabilities of occurrence *p_it _*were taken as identical at each time point. The different values considered for *p_it _*were 10% and 60%.

Only the first occurrence of the event was of interest. Thus, the final endpoint *E_i _*(occurrence/non-occurrence) was either non-occurrence of the event in subject *i *if all *E_it _*values (1≤ *t *≤4) were zeros, or occurrence of the event in subject *i *if one of the *E_it _*values was one. In other words, the final endpoint *E_i _*was a binary variable indicating whether a subject *i *experienced the event during follow-up.

The final true occurrence rate *P(E) *represented the cumulative occurrence rate of *E_i _*at the end of the follow-up, calculated in this complete cohort of 100 000 subjects.

### Attrition generation

The binary variable *D_it _*indicates whether subject *i *dropped out immediately after time *t*. If *D_ik _*= 1, then *X_ij _*and *E_ij _*are missing for all *j *>*k*. Note that drop-out can only occur after non-occurrence of the event at the previous time point(s), and that all variables were measured in all subjects at the first time point (i.e. no missing data on *X_i1 _*and *E_i1_*).

The variable *D_it _*(interrupt/continue) was drawn from a Bernoulli distribution with a probability of drop-out of *r_it_*, so that *r_it _*= *P(D_it _= 1)*. In order to make *X *a predictor of *D*, the value of *r_it _*was linked to the value of *X_it _*in the same way as for *p_it_*. Indeed, the probabilities of drop-out *r_it _*could differ according to the category of subjects, thus being linked to *X_it_*. This corresponds to the assumption that the probability of drop-out immediately after time point *t *depended on the observed covariate at time point *t*. For a given category of subjects, the probabilities of drop-out *r_it _*were taken as identical at each time point. The different values considered for *r_it _*were 10%, 30% and 60% (see Table 1).

**Table 1:** Probabilities of occurrence of the event and probabilities of drop-out at each time point, according to the subject category

Scenario n°	Probability of occurrence of the event *p_it _*at each time point (%)		Probability of drop-out *r_it _*after each time point (%)		Total drop-out rate (%)	Final true occurrence rate of the event *E *(%)
			
	Category *C_1_*	Category *C_2_*	Category *C_1_*	Category *C_2_*		
1	10	10	10	10	22.2	34.4
2	10	30	10	10	17.6	54.1
3	10	60	10	10	12.6	80.4
4	10	10	10	60	60.0	34.4
5	10	30	10	60	45.9	61.3
6	10	60	10	60	27.6	80.4
7	10	30	60	10	47.1	61.3
8	10	60	60	10	40.8	80.4

The total drop-out rate represented the cumulative drop-out rate at the end of the follow-up, calculated in the cohort of 100 000 subjects with drop-outs.

### Methods

The Kaplan-Meier method [5,6] was applied considering drop-out as censor. Subjects encountering four non-events were also censored at the end of the study. The event occurrence rate was estimated as [*1-S(t)*] at time *t *= 4, *S(t) *being the survival function.

Multiple imputation consists of replacing missing values with a set of plausible values, based on auxiliary information. In this way, all subjects can be included in the analysis. Multiple imputation was implemented with the SAS MI procedure (SAS Institute Inc. 2004. SAS/STAT^® ^9.1 User's Guide. Cary, NC: SAS Institute Inc.). Multiple imputation was performed at each time point (except the first, where all subjects were observed) for the subjects who failed and dropped out at a previous time point. For instance at time point 2, values were imputed for subjects i with *E_i1 _*= 0 (ie subjects with failure at time point 1); at period 3, values were imputed for subjects i with *E_i2 _*= 0 (ie subjects with failure at time point 2, regardless this failure is observed or imputed at the previous step). A specific SAS program executed the successive imputations one after another. The covariate *X_it _*was first imputed linearly according to the covariate and the outcome value at the previous time (*X_it-1 _*and *E_it-1 _*respectively). The response *E_it _*was then imputed by using logistic regression according to observed and imputed values of this covariate *X_it_*. The covariate *X_it _*was included linearly in the logistic regression to enable an evaluation of the results of the multiple imputation procedure as close as possible to its practical use, in which the threshold 0.014 is unknown. As only the first occurrence of the event was of interest, the response *E_it _*was imputed only when *E_ij _*= 0 for all *j *<*t*. Thus, the covariate *X_it _*and the intermediate outcome *E_it _*were imputed at each time point following drop-out of subject *i*, until the event occurred or until the end of follow-up. Then the final endpoint *E_i _*was calculated. Following Rubin's recommendations, five imputations were made at each time point [24].

### Design of the evaluation

Table 1 presents the eight scenarios that were tested and gives the final drop-out and true occurrence rates resulting from each of the eight scenarios. A scenario was determined by a probability of occurrence *p_it _*and a probability of drop-out *r_it _*for each category of subjects *C_1 _*and *C_2_*. Scenarios explored different contrasts between the categories *C_1 _*and *C_2_*, starting from scenario 1 which was the reference scenario where all probabilities equalled 10%. The robustness of the Kaplan-Meier method and the performance of multiple imputation were examined as the difference between the categories *C_1 _*and *C_2 _*was amplified.

In scenarios 2 and 3, the difference between the categories *C_1 _*and *C_2 _*lay only in the probabilities of occurrence *p_it_*. The probabilities of drop-out *r_it _*were equal in both categories (*r_it _*= 10% for all subjects). This means that drop-out did not depend on the observed value of the covariate *X *(nor on any other observed or unobserved value), and so that the data were missing completely at random (MCAR). In these cases, Kaplan-Meier and multiple imputation estimates were expected to be unbiased.

In scenarios 4 to 8, the probabilities of occurrence *p_it _*and the probabilities of drop-out *r_it _*differed according to the category (*p_it _*= 10%, 30%, 60%; *r_it _*= 10%, 60%). The difference increased to peak with the last scenario, in which probabilities of occurrence and drop-out corresponded to inverse situations between the categories. The difference between probabilities of drop-out means that drop-out depended on the observed value of the covariate *X*, and so that the data were missing at random (MAR). Scenario 4 was a particular case, where both categories had the same probability of occurrence of the event (*p_it _*= 10%). Consequently, even if probabilities of drop-out differed, both methods were expected to be unbiased. Otherwise, multiple imputation was expected to handle MAR attrition, whereas the Kaplan-Meier method was not. Scenarios 4 to 6 corresponded to the situation where the second category of subjects *C_2 _*had a higher chance of occurrence of the event *p_it _*and also a higher chance of drop-out *r_it_*. This could happen when the event is negative, such as a disease: patients more likely to be ill are more likely to drop out of the cohort. Scenarios 7 and 8 corresponded to the inverse situation where the second category of subjects *C_2 _*had a higher chance of occurrence of the event *p_it _*but a lower chance of drop-out *r_it_*. This could reflect the IVF situation, where women more likely to have a child are less likely to discontinue.

For each scenario, a cohort of 100 000 subjects was created with corresponding *p_it _*and *r_it_*. For the simulation study, 500 replications of samples of 2000 subjects were drawn from each simulated cohort of 100 000 subjects. The cumulative probability *P(E) *of the event *E *during follow-up was estimated in each sample using both methods (Kaplan-Meier and multiple imputation). For each scenario, mean value of the estimate and of the standard error of the 500 replications were computed. Bias and the coverage rate for the 95% confidence interval of *P(E) *were also provided. Bias was defined as the mean difference over the 500 replications between the estimation obtained by the method and the true occurrence rate of the event *E *in the complete simulated cohort (100 000 subjects with no drop-outs). The coverage rate was the percentage of times when the 95% confidence interval contained the true value of *P(E)*.

## Results

Results of the simulations for the 8 scenarios are displayed in Table 2.

**Table 2:** Estimation of the final occurrence rate of the event *P(E) *at the end of follow-up according to the estimation method in MCAR and MAR scenarios (500 replications of samples sized 2000)

	Scenario n°	True occurrence rate of the event *P(E) *(%)	Kaplan-Meier				Multiple Imputation			
				
			Estimate ^(a)^	Standard Error ^(a)^	Bias ^(b)^	Coverage ^(c)^	Estimate ^(a)^	Standard Error ^(a)^	Bias ^(b)^	Coverage ^(c)^
MCAR scenarios	1	34.4	34.3	1.1	-0.04	94.6	34.4	1.2	-0.01	94.6
	2	61.3	61.3	1.2	-0.09	95.6	61.3	1.2	-0.08	95.0
	3	80.4	80.5	1.0	0.08	95.8	80.5	1.0	0.09	95.4

MAR scenarios	4	34.4	34.3	1.5	-0.07	95.2	34.4	1.8	0.01	94.4
	5	61.3	56.3	1.6	-5.1	10.6	61.5	1.8	0.2	92.4
	6	80.4	75.7	1.2	-4.7	2.6	80.6	1.1	0.2	92.2
	7	61.3	66.2	1.6	4.9	14.8	63.0	1.8	1.6	85.2
	8	80.4	87.4	1.5	7.0	0.2	82.1	1.8	1.7	83.4

(a) mean value of the estimate and of the standard error of the 500 replications

(b) difference between estimate and the true occurrence rate *P(E)*

(c) proportion of 500 simulations-runs where the 95% Confidence Interval contained the true occurrence rate

As expected, in MCAR scenarios 1, 2 and 3, both methods (Kaplan-Meier and multiple imputation) were unbiased. Their coverage rates were close to the nominal level of 95% (range = [94.6%; 95.8%]).

In scenario 4, data were MAR but probabilities of occurrence of the event were equal in both categories of subjects. Both methods were as efficient as in MCAR scenarios: estimates were unbiased and coverage rates close to 95%. This was expected because both categories had the same probability of occurrence of the event (= 10%), so the difference in the probabilities of drop-out did not impact the estimation.

In the MAR scenarios 5 to 8, the amplitude of bias obtained with the Kaplan-Meier method was approximately 10% of *P(E) *(bias≈5%) and the coverage rate was very poor (less than 15%). The bias tended towards an underestimation of the true occurrence rate of the event *E *when subjects in the category *C_2 _*with a higher probability of the event had a higher probability of drop-out (scenarios 5 and 6), and towards an overestimation when subjects in the category *C_2 _*with a lower probability of the event had a higher probability of drop-out (scenarios 7 and 8). The bias was worse in the last scenario where the difference between the categories was maximised.

In these MAR scenarios 5 to 8, multiple imputation yielded a very small bias (less than 2%) and a very good coverage rate (above 83%). The method seemed to be less efficient in scenarios 7 and 8 when the category *C_2 _*with the higher probability of occurrence had the lower probability of drop-out.

**Figure 1:**
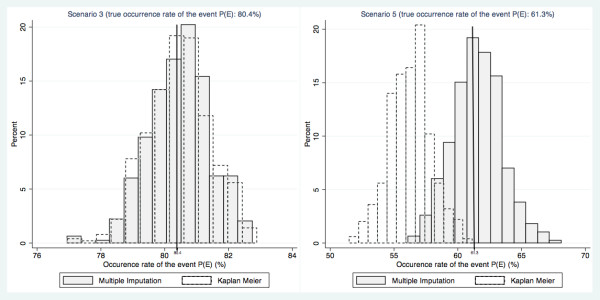
**Histograms of all estimates according to the method (multiple imputation and Kaplan Meier) for scenarios 3 (MCAR) and 5 (MAR)**.

To point up the importance of the difference between the estimations distributions in MAR scenarios, histograms of all estimates according to the method used are provided in Figure 1 for scenarios 3 (MCAR) and 5 (MAR).

Simulations were done with a smaller sample size of 500 (results not shown). This led to larger standard errors, that mechanically increased the coverage rate. In scenarios 5 to 8, the coverage rates ranged from 41.6% to 69.4% for the Kaplan-Meier estimate and from 88.4% to 94.8% for multiple imputation estimations. Bias was unchanged. Conclusions remained the same concerning the performance of the methods.

## Discussion

Multiple imputation has been developed to deal with missing data, which is a particular problem in longitudinal studies which suffer from attrition. However, little attention has been paid to how multiple imputation might be applied to studies with successive data collection points. The estimation of an occurrence rate in a cohort with attrition is a recurrent issue in reproductive health in general, and in IVF studies in particular. These studies have the characteristics of having discrete time points corresponding to attempts. Intervals between time points are not taken into account, thus leading to no distribution of the event times. Methods developed in previous publications thus cannot be applied here [25,26] We therefore used a simulation study to compare the performance of multiple imputation with that of the widely used Kaplan-Meier method, for the estimation of an occurrence rate in a cohort with attrition.

We found that in MAR scenarios, Kaplan-Meier estimation performed fairly well with respect to bias, but not with respect to the coverage rate. The direction of the biases was as expected, owing to the differences in the probabilities of occurrence of the event and of drop-out between the two subject categories. Indeed, when subjects dropping out tended to have a higher chance of experiencing the event, the Kaplan-Meier assumption that subjects dropping out have the same chance of occurrence of the event as other subjects led to an under-estimation, and vice-versa. The amplitude of bias was quite small. However, the Kaplan-Meier estimate was very precise around this biased estimation. This implies that the confidence interval rarely included the true value, and therefore that the coverage rate was poor. Thus, even if the bias is considered acceptable, one must be aware that the confidence interval will almost never contain the true value.

In contrast, multiple imputation was virtually bias-free and gave robust estimates in all the scenarios. This approach enables auxiliary information to be incorporated in the estimation. This makes it possible to take into account the fact that subjects' chances of experiencing the event may differ. This was the case in the simulated cohort in which two categories of subjects co-existed and were defined by their covariate values. Thus, multiple imputation succeeded in correcting the bias inherent in the Kaplan-Meier method. By replacing each missing value with several values, multiple imputation takes into account the uncertainty of the imputed value [23,27]. Therefore, the standard errors of multiple imputation were larger than those of the Kaplan-Meier estimation, resulting in larger confidence intervals and better coverage rates.

In comparison with the method of Hsu et al. [22], multiple imputation makes it possible to include the information contained in auxiliary variables without summarizing them in risk scores. Moreover, it gives a good estimation of variance, as it takes into account the uncertainty of the imputed values.

Multiple imputation was evaluated here under the assumption that the probability of drop-out immediately after time point *t *depended on the observed covariate at time point *t*. This type of attrition is likely to occur in most epidemiological cohorts. For example, a cohort of patients attending regular medical appointments would fit this attrition mechanism if the probability of dropping out after a given visit was likely to depend on the results of the same visit. This is the case in IVF, where failure of a procedure may indicate a poor chance of subsequent success and lead a couple to discontinue treatment. Multiple imputation at each time point is compatible with this attrition mechanism, as imputation at time point *t *is performed according to the observed or imputed covariate values at the same time point. This highlights the fact that data have to be investigated in order to apply a multiple imputation strategy that fits their pattern. The part of the auxiliary information that has to be included in the imputation model would be of particular interest: in some situations, baseline data could be sufficient, whereas in other situations, inclusion of intermediate measures could be advantageous. Imputation appears a preferable method than complete case analysis that assumes stronger hypotheses (MCAR data) and induces an important loss of statistical power.

The multiple imputation method could be generalized without theoretical problems to situations with a larger number of time intervals. However, it would rise practical difficulties such as a (much) longer computational time and a decreasing accuracy of the estimates, especially with limited sample size, due to categories of subjects with drop-out rates almost equal to 100% as the time goes by.

Data not missing at random were not tested in this work. In practice, implementations of multiple imputation in statistical software are made under the MAR assumption. Attrition was fully explained in the simulation study, as all the variables explaining attrition were taken into account. This may not be the case with observational data. Sensitivity analyses are necessary to determine whether the results are robust to deviations from this assumption.

The second difference between simulated and observational data is that, with simulated data, the model underlying the response and the covariates is known. The relations are then correctly used for imputation. With observational data,matters can be much more difficult. The relations used for imputation rarely reproduce exactly the relations underlying the data. With observational data, multiple imputation could lose precision and eventually generate bias. The consequences of misspecification of the imputation model were not explored here and require more research.

## Conclusions

Although our findings have the limitations of any simulation study, they point to a new way of estimating an occurrence rate in cohorts with attrition, using auxiliary information collected during follow-up. The robust estimations of multiple imputation in this simulation study are very satisfactory and promising. Our results suggest that in an observational context, comparison of the results of Kaplan-Meier estimation and of multiple imputation could provide a measure of the impact of attrition on the estimation. If this impact appears small, further analyses could be made using the easier-to-implement Kaplan-Meier approach.

This study is a first step towards defining the appropriate use of multiple imputation in longitudinal studies.

## Abbreviations

IVF: in vitro fertilization; MAR: missing at random; NMAR: not missing at random

## Competing interests

The authors declare that they have no competing interests.

## Authors' contributions

NS designed and implemented the simulation study, conducted the analysis and drafted the article. ELR and JB helped to design the study and interpret the results and they critically revised the article. All the authors read and approved the final manuscript.

## Pre-publication history

The pre-publication history for this paper can be accessed here:

http://www.biomedcentral.com/1471-2288/10/79/prepub

## References

[B1] Deeg DJ Attrition in longitudinal population studies: does it affect the generalizability of the findings? J Clin Epidemiol 2002 55 3 213 215 10.1016/S0895-4356(01)00472-3

[B2] Crawford SL Tennstedt SL McKinlay JB A comparison of analytic methods for non-random missingness of outcome data J Clin Epidemiol 1995 48 2 209 219 10.1016/0895-4356(94)00124-9 7869067

[B3] Greenland S Response and follow-up bias in cohort studies Am J Epidemiol 1977 106 3 184 187 900117 10.1093/oxfordjournals.aje.a112451

[B4] Pennefather PM Tin W Clarke MP Dutton J Fritz S Hey EN Bias due to incomplete follow up in a cohort study Br J Ophthalmol 1999 83 6 643 645 10.1136/bjo.83.6.643 10340968 PMC1723091

[B5] Kaplan EL Meier P Nonparametric estimation from incomplete observations J Am Stat Assoc 1958 53 282 457 481 10.2307/2281868

[B6] Altman DG Practical statistics for medical research 1991 London: Chapman & Hall

[B7] Van Beijsterveldt CE van Boxtel MP Bosma H Houx PJ Buntinx F Jolles J Predictors of attrition in a longitudinal cognitive aging study: the Maastricht Aging Study (MAAS) J Clin Epidemiol 2002 55 3 216 223 10.1016/S0895-4356(01)00473-5 11864790

[B8] McLean RR Hannan MT Epstein BE Bouxsein ML Cupples LA Murabito J Kiel DP Elderly cohort study subjects unable to return for follow-up have lower bone mass than those who can return Am J Epidemiol 2000 151 7 689 692 10752796 10.1093/oxfordjournals.aje.a010263

[B9] Daya S Life table (survival) analysis to generate cumulative pregnancy rates in assisted reproduction: are we overestimating our success rates? Hum Reprod 2005 20 5 1135 1143 10.1093/humrep/deh889 15790603

[B10] Olivius C Friden B Borg G Bergh C Why do couples discontinue in vitro fertilization treatment? A cohort study Fertil Steril 2004 81 2 258 261 10.1016/j.fertnstert.2003.06.029 14967352

[B11] Sharma V Allgar V Rajkhowa M Factors influencing the cumulative conception rate and discontinuation of in vitro fertilization treatment for infertility Fertil Steril 2002 78 1 40 46 10.1016/S0015-0282(02)03160-6 12095488

[B12] Olivius K Friden B Lundin K Bergh C Cumulative probability of live birth after three in vitro fertilization/intracytoplasmic sperm injection cycles Fertil Steril 2002 77 3 505 510 10.1016/S0015-0282(01)03217-4 11872203

[B13] Stolwijk AM Wetzels AM Braat DD Cumulative probability of achieving an ongoing pregnancy after in-vitro fertilization and intracytoplasmic sperm injection according to a woman's age, subfertility diagnosis and primary or secondary subfertility Hum Reprod 2000 15 1 203 209 10.1093/humrep/15.1.203 10611213

[B14] Rubin DB Multiple imputations in sample surveys - a phenomenological Bayesian approach to nonresponse Proc Survey Res Meth Sec (American Statistical Association) 1978 20 34

[B15] Barzi F Woodward M Imputations of missing values in practice: results from imputations of serum cholesterol in 28 cohort studies Am J Epidemiol 2004 160 1 34 45 10.1093/aje/kwh175 15229115

[B16] Wood AM White IR Thompson SG Are missing outcome data adequately handled? A review of published randomized controlled trials in major medical journals Clin Trials 2004 1 4 368 376 10.1191/1740774504cn032oa 16279275

[B17] Klebanoff MA Cole SR Use of multiple imputation in the epidemiologic literature Am J Epidemiol 2008 168 4 355 357 10.1093/aje/kwn071 18591202 PMC2561989

[B18] Kristman VL Manno M Côté P Methods to account for attrition in longitudinal data: do they work? A simulation study Eur J Epidemiol 2005 20 8 657 662 10.1007/s10654-005-7919-7 16151878

[B19] Engels JM Diehr P Imputation of missing longitudinal data: a comparison of methods J Clin Epidemiol 2003 56 10 968 976 10.1016/S0895-4356(03)00170-7 14568628

[B20] Mazumdar S Tang G Houck PR Dew MA Begley AE Scott J Mulsant BH Reynolds CF III Statistical analysis of longitudinal psychiatric data with dropouts J Psychiatr Res 2007 41 12 1032 1041 10.1016/j.jpsychires.2006.09.007 17092516 PMC2047601

[B21] Hsu CH Taylor JM Murray S Commenges D Survival analysis using auxiliary variables via non-parametric multiple imputation Stat Med 2006 25 20 3503 3517 10.1002/sim.2452 16345047

[B22] Hsu C-H Long Q Alberts D Estimation of colorectal adenoma recurrence with dependent censoring BMC Med Res Methodol 2009 9 1 66 10.1186/1471-2288-9-66 19788750 PMC2760573

[B23] Little RJ Rubin DB Statistical Analysis with Missing Data 2002 2 Hoboken, NJ: John Wiley & Sons Inc

[B24] Rubin DB Multiple imputation after 18+ years J Am Stat Assoc 1996 91 434 473 489 10.2307/2291635

[B25] Taylor JMG Murray S Hsu C-H Survival estimation and testing via multiple imputation Statistics & Probability Letters 2002 58 3 221 232

[B26] Jan B Shah S Shah S Fazli Qadir M Weighted Kaplan Meier estimation of survival function in heavy censoring Pak J Statist 2005 21 1 55 63

[B27] Schafer JL Analysis of Incomplete Multivariate Data 1997 72 Boca Raton, FL: Chapman & Hall/CRC

